# Molecular Landscape of Advanced Endometrial Cancer: Exploratory Analyses at Modena Cancer Center (MEMO)

**DOI:** 10.3390/ijms27021096

**Published:** 2026-01-22

**Authors:** Marta Pirola, Eleonora Molinaro, Samantha Manfredini, Riccardo Cuoghi Costantini, Chiara Carlucci, Claudia Piombino, Stefania Pipitone, Maria Giuseppa Vitale, Roberto Sabbatini, Francesca Bacchelli, Laura Botticelli, Albino Eccher, Roberto D’Amico, Lucia Longo, Stefania Bettelli, Cinzia Baldessari, Massimo Dominici

**Affiliations:** 1 Division of Oncology, University of Modena and Reggio Emilia, 41124 Modena, Italy; marta.pirola04@gmail.com (M.P.); 325498@studenti.unimore.it (C.C.); mdominici@unimore.it (M.D.); 2 Oncology Area Sud AUSL Modena, Ospedale of Sassuolo, 41049 Sassuolo, Italy; e.molinaro@ausl.mo.it (E.M.); l.longo@ausl.mo.it (L.L.); 3 Division of Molecular Pathology and Predictive Medicine, Azienda Ospedaliero-Universitaria of Modena, 41124 Modena, Italy; manfredini.samantha@aou.mo.it (S.M.); bettelli.stefania@aou.mo.it (S.B.); 4 Statistics Unit, Department of Diagnostic and Clinical Medicine and Public Health, University of Modena and Reggio Emilia, 41124 Modena, Italy; 337988@studenti.unimore.it (R.C.C.); roberto.damico@unimore.it (R.D.); 5 Division of Oncology, Department of Oncology & Hematology, Azienda Ospedaliero-Universitaria of Modena, 41124 Modena, Italy; claudia.piombino@outlook.com (C.P.); pipitone.stefania@aou.mo.it (S.P.); vitale.mariagiuseppa@aou.mo.it (M.G.V.); sabbrob@unimore.it (R.S.); 6 Clinical Trials Office, Division of Oncology, University of Modena and Reggio Emilia, 41124 Modena, Italy; francesca.bacchelli@unimore.it; 7 Department of Pathology, Azienda Ospedaliero-Universitaria of Modena, 41124 Modena, Italy; botticelli.laura@aou.mo.it (L.B.); eccher.albino@aou.mo.it (A.E.)

**Keywords:** endometrial cancer, molecular analysis, prognosis and treatments

## Abstract

Despite the introduction of novel therapeutic options, the prognosis of advanced endometrial cancer remains poor. In recent years, increasing attention has been directed toward the molecular characterization of endometrial cancer. However, data specifically focusing on advanced-stage disease are still limited. In our single-center, retrospective, exploratory study with a limited sample size, we analyzed 32 patients with advanced or recurrent endometrial cancer treated at the Modena Cancer Center. Comprehensive molecular profiling was performed to assess DNA mutations, copy number variations, and RNA expression. We characterized the molecular landscape of this cohort, evaluated selected genomic alterations across predefined clinical subgroups, and explored their association with overall survival. Consistent with previous reports, a high prevalence of *PTEN* and *PIK3CA* mutations were observed. Patients experiencing relapse more than six months after diagnosis were more likely to harbor *CTNNB1* mutations. *KRAS* mutations were more frequently detected in younger patients and in those with endometrioid histology, whereas *PPP2R1A* and *TP53* mutations were enriched in tumors with non-endometrioid histology. Notably, *CTNNB1* mutations were associated with a favorable prognostic impact, while *KRAS* mutations correlated with poorer overall survival. Our findings underscore the need for further investigation into the molecular landscape of advanced endometrial cancer, particularly in the context of therapeutic implications. Combinatorial treatment strategies targeting specific molecular alterations, such as *KRAS*, in combination with other targeted agents or therapeutic approaches, warrant further exploration.

## 1. Introduction

### 1.1. General Characteristics of Endometrial Cancer

**Figure 1:**
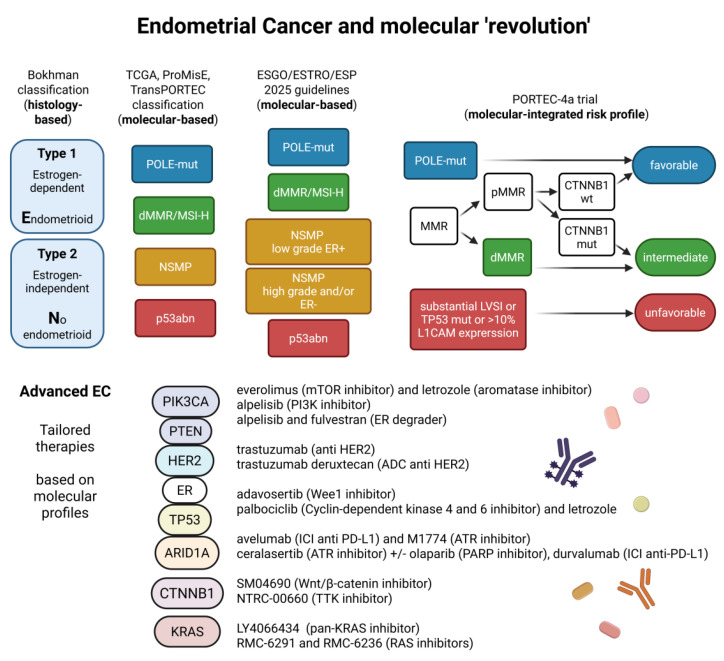
Evolution of endometrial cancer (EC) classification and potential clinical implications of molecular analysis in advanced EC. The upper part of the figure illustrates the evolution of endometrial cancer classification. From left to right: Bokhman’s Type I and II model, followed by the four-subgroup classifications proposed by TCGA, ProMisE, and TransPORTEC, and the five-group classification included in the 2025 ESGO/ESTRO/ESP guidelines. In the upper right, the integration of molecular and immunohistochemical analyses used in the PORTEC-4a trial is shown, dividing patients into three risk groups (favorable, intermediate, and unfavorable). The different colors of the subgroups show the differences visually (blue: better prognosis/less risk of recurrence, green and yellow: intermediate, red: worse). The lower part of the figure depicts selected potential therapeutic targets in advanced EC and the corresponding drugs, with the drug type indicated in parentheses. Created in BioRender.com (accessed on 14 December 2025).

Endometrial Cancer (EC) is the most common gynecologic malignancy in Western countries, and both its incidence and mortality rates are steadily increasing. This upward trend is expected to continue over the next few decades [1]. More than 90% of EC cases are sporadic [2]. Although patients diagnosed with early-stage EC generally have a favorable prognosis, approximately 15–20% present with high-risk disease. This is associated with a higher likelihood of distant metastases and cancer-related mortality [3]. Outcomes for patients with advanced or recurrent EC remain poor [2]. Historically, EC has been classified into two major subtypes based on clinical, endocrine, and metabolic features, according to the Bokhman model [4]. Over the past decade, a major advancement in the diagnosis and management of EC has been the introduction of molecularly driven classification systems. In 2013, The Cancer Genome Atlas (TCGA) published a comprehensive molecular characterization of EC [5]. Subsequently, the Proactive Molecular Risk Classifier for Endometrial Cancer (ProMisE) [6] and TransPORTEC consortium [3] proposed a four-subtype molecular classification that is more readily applicable in routine clinical practice (Figure 1):

- *POLE*-ultramutated (*POLE*mut): characterized by mutations in the DNA polymerase epsilon gene, with an excellent prognosis.
- Mismatch repair-deficient (dMMR)/microsatellite instability-high (MSI-H): defined by loss of mismatch repair function and high levels of microsatellite instability.
- p53-abnormal (p53abn): characterized by *TP53* mutations and aberrant p53 protein expression; with aggressive clinical behavior.
- No specific molecular profile (NSMP): tumors lacking the defining features of the other subtypes.

The 2025 ESGO/ESTRO/ESP guidelines incorporated the revised FIGO staging system for EC [7] as well as the growing body of evidence informing EC management. Their aim was to improve the definition of prognostic groups and to identify clinically relevant treatment subgroups, with particular emphasis on the NSMP category. The NSMP subtype accounts for approximately 50% of EC cases in population-based cohorts, prompting increasing interest in the identification of additional biomarkers to refine risk stratification within this heterogeneous group. Among these, estrogen receptor (ER) expression and L1 neuronal cell adhesion molecule (L1CAM) have emerged as promising candidates for prognostic refinement [8].

Accordingly, the 2025 ESGO/ESTRO/ESP guidelines stratify NSMP tumors into low-grade ER-positive or high-grade and/or ER-negative subgroups [7]. Vermij et al. [9] evaluated whether alterations in the ER, progesterone receptor (PR), L1CAM, and β-catenin 1 (*CTNNB1*), together with other clinico-pathological and molecular risk factors, could enhance EC risk assessment. High-risk EC patients from the PORTEC-3 trial and the Medisch Spectrum Twente (MST) prospective cohort were analyzed. Although ER, PR, L1CAM, and *CTNNB1* did not demonstrate independent prognostic value, ER positivity was associated with a favorable prognosis within the NSMP subgroup [9]. Furthermore, in a large cohort of EC patients treated at the University Hospital Tübingen, L1CAM expression significantly stratified risk among tumors belonging to the p53 wild-type/NSMP ProMisE subgroup [10].

β-catenin and L1CAM exhibit a mutually exclusive expression pattern [11]. Both molecules play key roles in the regulation of cell proliferation, adhesion, and migration. In the study by Yoon et al., β-catenin positivity on immunohistochemistry (IHC) was identified as a prognostic factor associated with worse progression-free survival (PFS) in the high-intermediate risk subgroup of 335 EC patients who underwent surgery at Seoul National University Bundang Hospital [11]. Similarly, abnormal β-catenin expression assessed by IHC was associated with vaginal recurrence in the NSMP subgroup in the study by Hui and colleagues [12]. In that trial, adjuvant radiotherapy in patients with stage I–II NSMP EC and abnormal β-catenin expression resulted in improved local control. Although abnormal nuclear β-catenin expression detected by IHC does not invariably correlate with *CTNNB1* mutations, it may serve as a useful surrogate marker for predicting their presence [13,14].

The PORTEC-4a trial is an ongoing prospective, multicentre, phase III study enrolling patients with high-intermediate risk EC. Participants are randomized to either an experimental arm, in which adjuvant treatment is guided by a molecular-integrated risk profile, or a standard arm receiving adjuvant vaginal brachytherapy. Molecular classification is based on IHC assessment of L1CAM, p53, and MMR status, combined with DNA sequencing for *POLE* and *CTNNB1* mutations. Three recurrence-free survival risk categories are defined: favorable (*POLE*-mutated or pMMR with *CTNNB1* wild-type (WT)), intermediate (pMMR with *CTNNB1*-mutation or dMMR), and unfavorable (substantial LVSI, p53-mutation, or >10% L1CAM-expression) (Figure 1). The primary endpoint of the trial is vaginal recurrence [15]. Preliminary findings indicate that molecular profiling enables personalization of adjuvant treatment according to individual patient risk [15], while final results are still awaited.

### 1.2. Therapeutic Options in Advanced EC: Present and Future, and the Relevance of the Molecular Landscape

Until recently, molecular classification had a limited impact on the management of advanced or recurrent EC, for which chemotherapy or hormone therapy represented the standard treatment approaches [16,17,18]. More recently, immune checkpoint inhibitors (ICI) in combination with chemotherapy have demonstrated promising efficacy as first-line treatment options [19,20,21], particularly in patients with dMMR or MSI-H. Nevertheless, therapeutic options for other molecular subgroups and for use beyond the first-line setting remain limited. Olaparib has been investigated as a maintenance strategy in combination with ICI following first-line chemotherapy plus immunotherapy [22]. In addition, pembrolizumab or dostarlimab monotherapy has been approved for patients with recurrent or advanced MSI-H EC who have previously received platinum-based chemotherapy [23,24]. For patients with previously treated advanced disease and pMMR, the combination of lenvatinib and pembrolizumab has demonstrated improved clinical outcomes [25,26].

**Figure 2:**
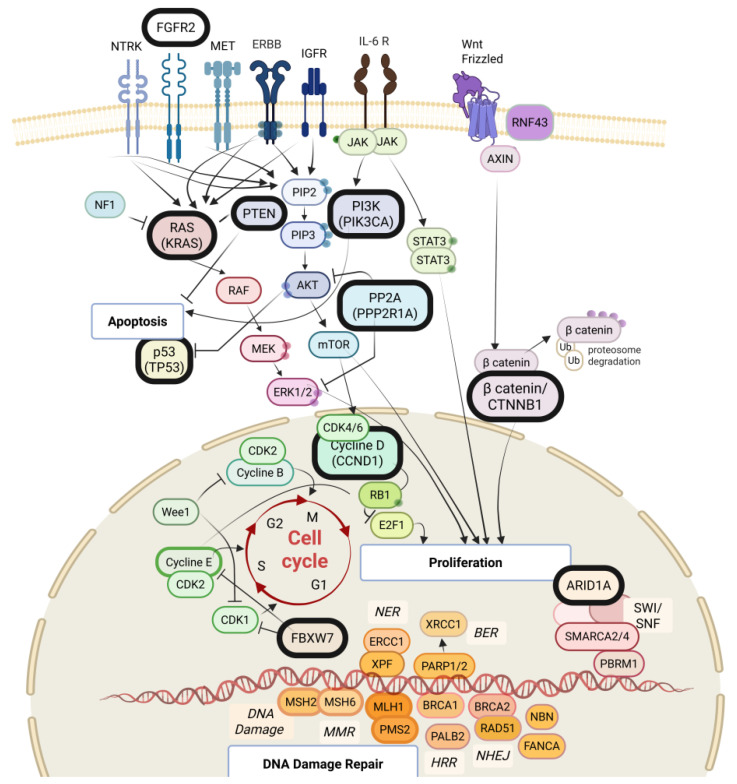
Molecular landscape of endometrial cancer, highlighting selected mutations. The 10 mutations selected for subsequent analyses are highlighted in black with a thick border, within the broader molecular landscape of endometrial cancer, emphasizing the integration and interdependence of key signaling pathways. Although not exhaustive, this representation provides a conceptual overview of the molecular context and potential interactions relevant to tumor biology. Created in BioRender.com (accessed on 29 November 2025).

Despite advances in adjuvant treatment strategies driven by molecular classifications, as well as the development of novel therapeutic options for advanced disease, a substantial proportion of patients continue to experience disease recurrence or progression. Consequently, personalized therapeutic approaches, including targeted agents and immunotherapy tailored to specific molecular alterations, are actively being explored [27] (Figure 1 and Figure 2).

Alterations in the phosphatidylinositol 3-kinase (PI3K) signaling pathway are frequent in EC, and *PIK3CA* mutations have been associated with poorer survival outcomes [28]. Several clinical trials have evaluated agents targeting the PI3K pathway, including the mammalian target of rapamycin (mTOR) inhibitors, either as monotherapy or in combination regimes. Notably, the combination of everolimus and letrozole achieved a high clinical benefit rate in patients with recurrent EC [29]. In 2024, Passarelli et al. reported clinical activity of the PI3K inhibitor alpelisib in patients with advanced gynecologic malignancies harboring *PIK3CA* mutations [30]. A phase II study investigating alpelisib in combination with fulvestrant in patients with *PIK3CA*-mutated, ER-positive endometrioid EC is currently ongoing (GOG-3069; NCT05154487).

HER2-targeted strategies have also shown efficacy in selected EC subgroups. A randomized phase II trial demonstrated improved outcomes with the addition of trastuzumab to carboplatin and paclitaxel in patients with HER2/neu-positive EC [31]. Furthermore, in the EC cohort of the Destiny-PanTumor02 trial, trastuzumab deruxtecan elicited clinically meaningful responses and survival benefit in patients with HER2-expressing locally advanced or metastatic disease who had received at least one prior systemic treatment or had no alternative treatment options [32].

Additional targeted approaches are under investigation. The WEE1 inhibitor adavosertib has demonstrated clinical benefit in heavily pretreated EC patients [33]. Preliminary results from the phase II ENGOT EN3 PALEO trial in hormone-positive, previously treated EC showed that the combination of letrozole and palbociclib significantly improved PFS compared with letrozole plus placebo [34,35]. Moreover, a phase II study is enrolling patients with recurrent EC harboring AT-rich interactive domain-containing protein 1A (*ARID1A*) mutations and prior exposure to immunotherapy, evaluating avelumab in combination with the ATR inhibitor M1774 (NCT06518564). The ATARI trial is further exploring the ATR inhibitor ceralasertib, alone or in combination with the PARP inhibitor olaparib or the anti-PD-L1 durvalumab, in patients with recurrent gynecologic cancers characterized by *ARID1A* loss-of-function mutations (NCT04065269).

Emerging evidence supports the targeting of *CTNNB1* alterations in EC. Moroney et al. demonstrated that SM04690, an inhibitor of the Wnt/β-catenin pathway, reduced cell viability in cell lines, with β-catenin transcriptional inhibitors showing particular efficacy in *CTNNB1*-mutated tumors [36]. Similarly, the threonine-tyrosine kinase (TTK) inhibitor NTRC-00660 suppressed tumor growth in a *CTNNB1*-mutant xenograft model. TTK inhibitors disrupt spindle assembly checkpoint signaling, leading to premature anaphase entry, mitotic catastrophe, and cell death. These findings suggest that *CTNNB1* mutations may serve as a predictive biomarker for response to TTK inhibitors in future clinical trials [37]. In addition, Fatima and colleagues identified five small-molecule inhibitors with potential activity against *CTNNB1*-driven EC, including pazopanib, binimetinib, telatinib, 4-(2,3-dihydrobenzo[b][1,4]dioxin-6-yl)-3-((5-nitrothiazol-2-yl)thio)-1H-1,2,4-triazol-5(4H)-one, and ribavirin, providing a foundation for further preclinical and translational studies [38]. Interestingly, the aforementioned everolimus–letrozole study also reported favorable responses in patients with endometrioid EC harboring *CTNNB1* mutations [29].

The prognostic and therapeutic implications of Kirsten Rat Sarcoma virus (*KRAS*) alterations in EC remain controversial. Birkeland et al. reported that *KRAS* gene amplification and over-expression, rather than mutations, were associated with aggressive and metastatic EC [39]. Conversely, Kilowski and colleagues suggested that *KRAS*-mutated EC represents a distinct molecular genotype, with partial overlap with genomic features predictive of immunotherapy response, such as high tumor mutational burden and MSI-high status, supporting the potential for biomarker-driven combination strategies [40]. Some clinical trials are currently evaluating *KRAS*-targeted therapies in solid tumors. A phase Ia/Ib study is investigating the pan-*KRAS* inhibitor LY4066434, alone or in combination with other agents, in patients with *KRAS* mutant solid tumors, including EC (NCT06607185). Additionally, an open-label Phase I trial is assessing the safety and preliminary antitumor activity of TCR-engineered T cells targeting *KRAS* mutations in patients with unresectable, advanced, or metastatic solid tumors (NCT06218914). Another study, NCT06128551, is evaluating the safety, tolerability, and pharmacokinetic (PK) of RMC-6291 and RMC-6236 in adults with *KRAS* G12C-mutated solid tumors. Finally, Ring et al. proposed that combining MEK inhibition with anti-estrogen therapy may enhance response rates in patients with *KRAS*-mutant EC [41].

### 1.3. Aim of the Study

The aim of our exploratory analyses is to evaluate the molecular landscape in advanced and recurrent EC by examining the association between patient subgroups and clinical and molecular variables, as well as the relationship between patient subgroups and specific genetic alterations in terms of survival outcomes. The identification of targetable mutations that are correlated with prognosis may contribute to the expansion of therapeutic strategies for advanced EC.

## 2. Results

### 2.1. Patients’ Characteristics

Of the 32 patients enrolled in the study, tumor samples suitable for mutational DNA analysis were available for 29 patients. Among these, 48.3% were diagnosed with stage IV disease at presentation or experienced disease relapse within six months of diagnosis (group M), whereas the remaining patients relapsed more than six months after diagnosis (group R). The median age at the time of relapse or metastatic disease was 72 years, with 51.7% of patients aged above this threshold (group O). Endometrioid histology was observed in 58.6% of cases (group E). According to the World Health Organization (WHO) criteria, 56.0% of patients were classified as overweight or obese, defined as a body mass index (BMI) ≥ 25 kg/m^2^ for overweight and ≥30 kg/m^2^ for obesity (Table 1).

### 2.2. Molecular Landscape of Advanced EC at Modena Cancer Center

#### 2.2.1. Mutational DNA Analysis

A total of 29 patient samples were evaluable for mutational DNA analysis. In three of the 32 enrolled cases, the available material was not suitable for analysis due to inadequate sequencing quality (Mapped reads < 300,000 and/or Library Mean Length < 75 bp and/or uniformity < 90%). Overall, 218 DNA variants were identified, of which 83 were present in publicly available cBioPortal datasets, corresponding to 201 distinct variants, including 69 reported by cBioPortal. The majority of variants were missense mutations and were classified as follows: 49 pathogenic (pa) (44 reported in cBioPortal), 57 likely pathogenic (lp) (32 reported in cBioPortal), 83 Variant of Uncertain Significance (VUS) (7 reported in cBioPortal), 12 likely benign, and 17 benign. The median number of mutations per patient was 7 (3 reported in cBioPortal).

Genes harboring more than 10 mutations included *PTEN* (24 variants: 16 pa, 7 lp, and 1 VUS), detected in 16 patients with a median allele frequency (mAF) of 31.3%; *PIK3CA* (16 variants: 10 pa, 5 lp, and 1 VUS), identified in 13 patients (mAF 30.3%); *ATM* (12 variants); and *ARID1A* (12 variants). *TP53* mutations were detected in 6 patients (all pa, 5 reported in cBioPortal), with a median allele frequency of 42.2%. Mutations in *NOTCH1*, *NOTCH2*, and *NOTCH3* were identified in 8 patients (10 variants in total: 4 lp, 4 VUS, and 2 benign), with a median allele frequency of 29.6%.

**Figure 3:**
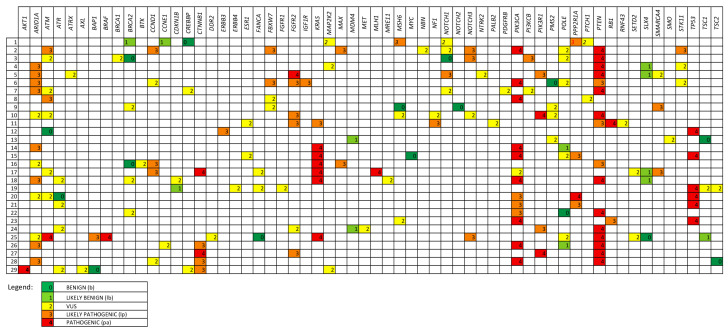
Molecular landscape of our population considering DNA variants identified in evaluable samples, annotated on the basis of cBioPortal and OncoKB datasets, and reclassified according to ACMG.

Figure 3 illustrates the molecular landscape in our study population, including all variants identified in evaluable samples, annotated using cBioPortal and OncoKB datasets, and reclassified for pathogenicity according to American College of Medical Genetics and Genomics (ACMG) criteria.

#### 2.2.2. Copy Number Variation DNA Analysis

Copy Number Variation (CNV) analysis was successfully performed in twenty-five samples. The remaining seven samples did not meet quality control criteria due to insufficient sequencing metrics (Mapped reads < 100,000 and/or Median Absolute Pairwise Difference (MAPD) > 0.5 and/or 95% Confidence Interval less than four copy numbers). Only one copy number alteration was identified in the cohort, consisting of an amplification of the *AKT2* gene, with a copy number of 6.45.

#### 2.2.3. RNA Analysis of Fusion Transcripts

RNA analysis for the detection of fusion transcripts was successfully performed in twenty-six samples. The remaining six samples failed to meet quality control criteria due to inadequate sequencing parameters (Mapped reads < 5000 and/or Library Mean Length < 60 bp and/or Minimum reads for at least three of five internal controls < 15). No RNA fusion events were identified in our study population.

### 2.3. Mutational Landscape in Different Groups of Patients

Within the molecular landscape of our cohort, we selected ten gene mutations (Figure 2 and Table 2) based on their potential clinical and biological relevance in advanced EC, as well as their possible therapeutic implications.

#### 2.3.1. Patients with Metastatic Disease at Onset or Relapsed Within 6 Months (M) Versus Patients Relapsing After 6 Months (R)

**Figure 4:**
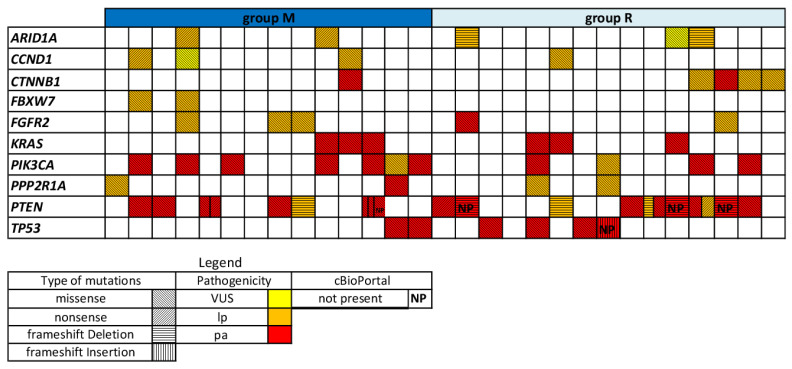
Graphical representation of the 10 selected mutations (cBioPortal-annotated and ACMG-classified) in group M (patients with metastatic disease at onset or relapse within 6 months of diagnosis) and group R (patients relapsing more than six months after diagnosis). Mutation pathogenicity is indicated by color: pathogenic (red), likely pathogenic (orange), and variants of uncertain significance (VUS, yellow). Mutation types are distinguished by background pattern: missense, nonsense, frameshift deletions, and frameshift insertions. Alterations not present in the cBioPortal dataset are indicated as ‘not present’ (NP).

Figure 4 presents a graphical comparison of selected filtered mutations—annotated in cBioPortal and classified as pathogenic according to ACMG criteria—between groups M and R. Table 3 summarizes the mutation types, frequency, and percentages across the entire cohort, as well as within each group.

Group R exhibited a higher likelihood of harboring pathogenic or likely pathogenic *CTNNB1* mutations Probability of direction (PD) 0.930; Region of Practical Equivalence (ROPE) 0.050), although the posterior distribution was highly variable (median log(OR) 1.75, 90% CI: −0.19 to 4.43). This group also had a greater probability of presenting with a higher BMI (median log (OR) 1.54, 90% CI: 0.15 to 3.06; PD 0.963; ROPE 0.029). Detailed results are provided in the Supplementary Material (Supplemental Table S1).

#### 2.3.2. Older Patients (O) Versus Younger Ones (Y)

Group Y showed a higher likelihood of carrying pathogenic or likely pathogenic mutations in *KRAS* (median log (OR) −2.18; 90% CI: −4.57 to −0.38; PD 0.9823; ROPE 0.008) and *PIK3CA* (median log (OR) −1.73; 90% CI: −3.31 to −0.40; PD 0.987; ROPE 0.000). In contrast, group O was more likely to harbor pathogenic or likely pathogenic *PTEN* mutations (median log (OR) 1.33; 90% CI: 0.07 to 2.68; PD 0.963; ROPE 0.044), with a trend towards higher prevalence of *FGFR2* pathogenic or likely pathogenic mutations (median log (OR) 1.80; 90% CI: −0.11 to 4.44; PD 0.937; ROPE 0.046). Detailed results are provided in the Supplementary Material (Supplemental Table S1).

#### 2.3.3. Patients with Endometrioid Histology (E) Versus Patients with Non-Endometrioid Histology (N)

Group E had a higher probability of carrying pathogenic or likely pathogenic *PTEN* mutations (median log(OR) −1.38; 90% CI: −2.78 to −0.12; PD 0.962; ROPE 0.038) and a greater likelihood of *KRAS* pathogenic or likely pathogenic mutations (PD 0.942; ROPE 0.052), although the posterior distribution was highly variable (median log(OR) −1.68; 90% CI: −4.20 to 0.06). Conversely, group N was more likely to harbor pathogenic or likely pathogenic mutations in *PPP2R1A* (PD 0.939; ROPE 0.045) and *TP53* (PD 0.927; ROPE 0.058), with high variability in the posterior distribution. Full results are provided in Supplemental Table S1.

#### 2.3.4. Overweight or Obese Patients (W) Versus Skinny Ones (S)

No strong evidence was observed for differences in the probability of selected mutations between group W and group S. However, group W was more likely to present with endometrioid histology (PD 0.947; ROPE 0.047), despite high variability in the posterior distribution (median log (OR) 1.36; 90% CI: −0.04 to 2.87). Complete analyses are reported in the Supplementary Materials (Supplemental Table S1).

### 2.4. Survival Analysis

Among the 29 patients with available molecular data, the median follow-up time from relapse or diagnosis of metastatic disease was 59.3 months.

In univariate analysis, patients who relapsed more than six months after diagnosis (group R) showed a trend toward improved survival, although the evidence was not strong (β median 0.84; 90% CI: −0.01 to 1.79; ROPE 0.042; PD 0.948). None of the other clinical subgroups demonstrated a statistically robust correlation with survival. Notably, patients harboring pathogenic or likely pathogenic *CTNNB1* mutations exhibited better survival (β median 1.42; 90% CI: 0.17 to 3.01; ROPE 0.015; PD 0.972). Whereas patients with pathogenic or likely pathogenic *KRAS* mutations had poorer prognosis (β median −1.72; 90% CI: −2.54 to −0.62; ROPE 0.000; PD 0.993). Detailed results for all univariate analyses are provided in the Supplementary Material (Supplemental Table S2).

**Figure 5:**
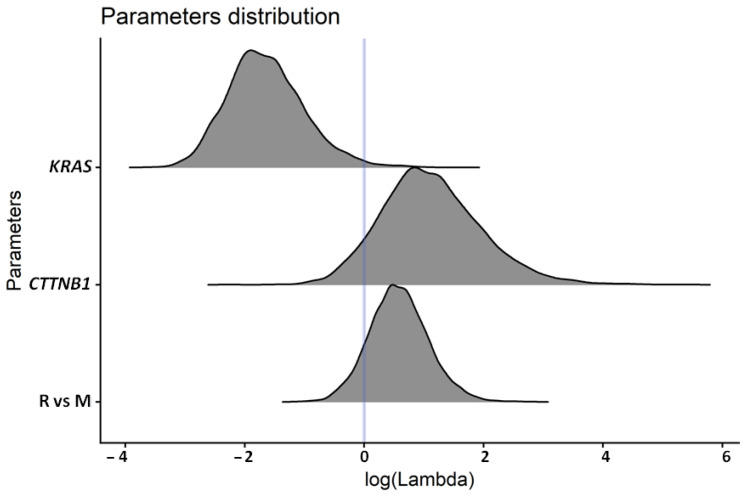
Graphical representation of survival distribution according to molecular and clinical subgroups. Pathogenic or likely pathogenic *KRAS* mutations were associated with poor survival, whereas pathogenic or likely pathogenic *CTNNB1* mutations were associated with more favorable outcomes, although with a weaker association. Survival distributions were also compared between patients relapsing more than 6 months after diagnosis (R) and those with metastatic disease at diagnosis or early relapse (M); this comparison did not retain statistical significance.

**Figure 6:**
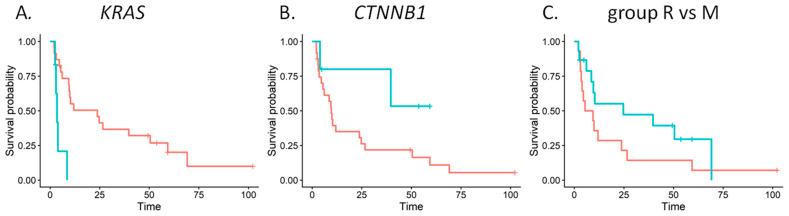
Kaplan–Meier survival curves for patients with pathogenic or likely pathogenic *KRAS* mutations (blue line) or without these mutations (red line) (**A**), with pathogenic or likely pathogenic *CTNNB1* mutations (blue line) or without (red line) (**B**) and patients relapsed after 6 months from diagnosis (blue line) or metastatic at diagnosis or relapsed before 6 months (red line) (**C**).

In multivariate analysis, pathogenic or likely pathogenic *KRAS* mutations remained strongly associated with poor survival (β median −1.69; 90% CI: −2.61 to −0.51; ROPE 0.000; PD 0.987). Pathogenic or likely pathogenic *CTNNB1* mutations remained associated with better survival, although the strength of the association was lower (β median 1.06; 90% CI −0.15 to 2.60; ROPE 0.041; PD 0.925). In contrast, relapse after 6 months lost statistical significance (β median 0.57; 90% CI: −0.21 to 1.42; ROPE 0.085; PD 0.889). The distributions of multivariable survival model parameters are presented in Figure 5. Kaplan–Meier survival curves for *KRAS*, *CTNNB1*, and group R versus M are shown in Figure 6.

## 3. Discussion

In our exploratory analysis, limited by the small sample size, retrospective nature, and patient characteristics’ heterogeneity, DNA mutational profiling proved feasible; whereas, copy number variation (CNV) analysis and RNA fusion transcript detection were more challenging due to quality limitations, particularly in older samples. Given the potential prognostic and therapeutic relevance, performing these analyses earlier in patients’ disease course—especially in aggressive or higher risk cases—may help guide clinical decision-making and optimize personalized treatment strategies. This approach is particularly pertinent in advanced EC, where targeted therapeutic options are expanding (Figure 1), yet prognosis remains poor.

Our molecular profiling corroborated previous findings, including the high frequency of alterations in the PI3K/PTEN pathway. Likely pathogenic or pathogenic mutations in *PIK3CA* were observed in 37.9% of patients, while over 50% harbored *PTEN* mutations, with 22 out of 29 patients exhibiting mutations in *PIK3CA* and/or *PTEN*. These alterations may have clinical significance, as demonstrated in studies investigating PI3K-targeted therapy such as alpelisib [30].

*ARID1A* mutations were detected in 13.8% of patients; this gene regulates chromatin accessibility, DNA repair, and gene expression, and its loss-of-function mutations contribute to genomic instability—a feature that may be therapeutically exploitable [36,44]. In EC, it often coexists with *PTEN*, *PIK3CA*, or MMR mutations [48,49]. In our cohort, *ARID1A* mutations co-occurred with *PIK3CA* (2 cases), *PTEN* (1 case), or both (1 case).

Pathogenic *TP53* mutations were identified in more than 20% of patients, all with high allele frequencies. As *TP53* mutations are driver events in EC, their prevalence underscores the high-risk nature of our cohort, which included only metastatic or relapsed cases. *TP53* abnormality defines a distinct molecular subgroup in the TCGA/ProMisE classification, characterized by genomic instability, aggressive behavior, and poor prognosis [6,51]. Although no therapies directly target *TP53*, indirect approaches—such as WEE1 inhibitors exploiting synthetic lethality (NCT03668340) or ATR/CHK1 inhibitors (ATARI trial)—are under investigation.

*KRAS* mutations were observed in 20.7% of our patients and always co-occurred with other mutations, complicating the assessment of their independent impact.

We analyzed correlations between clinical features (age, BMI) and tumor characteristics (relapse timing, histotype) with selected DNA mutations.

Patients relapsing after six months (group R) were more likely to harbor pathogenic or likely pathogenic *CTNNB1* mutations, consistent with prior reports describing higher *CTNNB1* mutation frequency in low-grade endometrioid EC, lower frequency in high-grade or serous EC, and association with an increased risk of relapse [44,52]. Group R also tended to have higher BMI, suggesting interactions between body composition and tumor biology.

Younger patients (<72 years) and those with endometrioid histology were enriched for *KRAS* mutations, whereas non-endometrioid tumors were more likely to harbor pathogenic or likely pathogenic *PPP2R1A* and *TP53* mutations, in line with the previous literature [5,47,53].

No strong evidence linked specific mutations to overweight or obesity, although higher BMI correlated with endometrioid histology, reflecting the well-established association between obesity and endometrioid EC risk [5,54,55,56].

Although our survival analysis indicated a trend towards better outcomes in patients with *CTNNB1* mutations, the small sample size, retrospective nature of the trial, and heterogeneous composition and treatment history of the cohort limit definitive conclusions. *CTNNB1* mutations may influence EC biology through pathways involving squamous differentiation, immune evasion, and calcium homeostasis regulation [57], and they are more frequent in low-grade tumors, a pattern that may persist in advanced EC [58]. Interactions with other molecular alterations, such as *POLE* or MMR mutations, could further modulate prognosis and response to immunotherapy [58]. Penick et al. described that endometrioid EC patients with high levels of *CTNNB1* transcript expression (rather than mutation) had a better outcome following adjuvant chemotherapy [59]. In our cohort, three of five patients with likely pathogenic or pathogenic *CTNNB1* mutations received pembrolizumab and lenvatinib in addition to one or more lines of chemotherapy, and all patients underwent chemotherapy. On the other hand, only one patient without likely pathogenic or pathogenic *CTNNB1* mutations was treated with the combination of pembrolizumab and lenvatinib in addition to chemotherapy, and seven patients received only best supportive care and/or local treatments. In short, *CTNNB1*-mutated patients received more lines and types of systemic therapies. This heterogeneity in treatment history may have contributed to the observed survival benefit in *CTNNB1*-mutated patients. The potential for selection bias and context-dependent effects must be acknowledged. Prior studies, including those from PORTEC-3 and MST cohorts, found no independent prognostic value of ER, PR, L1CAM, or *CTNNB1* in high-risk EC [10].

Conversely, likely pathogenic or pathogenic *KRAS* mutations were associated with a poorer prognosis in our cohort. Once again, the aforementioned limitations restrict the interpretability and generalizability of this result. While *KRAS* amplifications have been linked to aggressive EC phenotypes [39], the prognostic significance of *KRAS* mutations remains inconsistent across studies. More aggressive clinical behavior was reported in *KRAS*-mutated postmenopausal endometrioid EC patients aged over 60 in a Japanese trial. This study involved only 4.5% of stage IV EC patients [60]. Conversely, a European trial concluded that *KRAS* mutations were relatively common in stage I–III EC but had no clear prognostic value [61]. The TCGA molecular classification emphasizes that the prognosis for EC depends primarily on the overall molecular subgroups, rather than a single mutation. Specifically, *KRAS* alone does not define a different prognosis. Additionally, no significant age-related differences are reported if there are no other critical alterations, such as *TP53* or *POLE* [5]. However, in other cancers, such as colon cancer, the interaction between *KRAS* status, prognosis, and age group was significant, and the prognosis of *KRAS* status was impacted by age group. *KRAS* mutation was a negative prognostic factor for survival among patients under 50 years old and between 50 and 69 years old, but lost its prognostic impact among those 70 years old and over [62]. EC patients with *KRAS* mutations may have a distinct genotype that overlaps with the genomic predictors of immunotherapy response [40]. None of our six patients with likely pathogenic or pathogenic *KRAS* mutations underwent immunotherapy, which may have affected our negative outcome results. The median age of patients with *KRAS* mutations in our population is 64 years, lower than the median age of the entire population (72 years). Constitutive activation of MAPK/ERK and PI3K/AKT signaling in *KRAS*-mutated tumors may promote tumor growth, invasion, and therapy resistance. Experimental evidence suggests that MEK inhibition combined with anti-estrogen therapy could enhance response rates in *KRAS*-mutant EC [41]. However, none of our patients received MEK inhibitors alone or in combination with anti-estrogen therapy.

Overall, the prognostic impact of individual mutations, including *KRAS* and *CTNNB1*, is context-dependent and influenced by co-occurring genomic alterations. The small sample size and retrospective nature of our study limit the generalizability of our findings, and mechanistic explanations remain speculative. Functional assays, orthogonal validation, and larger prospective trials are needed to confirm whether these mutations and their associated pathways influence treatment response or survival. Further preclinical and clinical investigations into targeted therapies, particularly those addressing KRAS and other actionable alterations, are warranted to improve outcomes in advanced EC.

## 4. Material, Patients, and Methods

### 4.1. Patient Enrolment and Sample Collection

We conducted a retrospective, single-center study. Thirty-two patients with histologically confirmed advanced or recurrent EC, diagnosed between 2008 and 2020 at Modena Cancer Center, Azienda Ospedaliero-Universitaria (AOU)—Policlinico of Modena, were included. A total of 32 formalin-fixed, paraffin-embedded tumor tissue samples were included for analysis. Histological subtypes, including uterine sarcomas, neuroendocrine tumors, and mesonephric-like EC, were excluded.

Among the 29 patients who underwent tumor DNA mutational analysis, tumor specimens were obtained predominantly from the primary tumor at diagnosis, including 18 surgical samples and nine diagnostic biopsy specimens. The remaining two samples were derived from metastatic lesions collected during systemic treatment (one after one line of chemotherapy and one after two lines of chemotherapy). Most patients received systemic chemotherapy (one or more lines); four patients were treated with chemotherapy (one or more lines) and with combinations of pembrolizumab and lenvatinb, while seven patients received best supportive care and/or local treatments.

### 4.2. Ethical Approval

The study protocol was reviewed and approved by the Area Vasta Emilia Nord Ethics Committee (protocol number: 36603/2020, approval date: 15 December 2020). It was conducted according to the Declaration of Helsinki principles. Written informed consent was obtained from alive and traceable patients.

### 4.3. Details of Molecular Analysis

Genomic DNA was extracted using a total DNA extraction kit following the manufacturer’s instructions from an area selected in the 32 formalin-fixed paraffin samples of EC. The analysis was performed using the Oncomine Comprehensive Assay^TM^ V3 (Thermofisher Scientific, Waltham, MA, USA), which comprises 161 genes, separated into 87 hotspot genes, 49 with full exon coverage, 43 copy number genes, and 51 gene fusions. All libraries’ preparation was performed according to the manufacturer’s protocol. Multiplex PCR amplifications were conducted using a DNA concentration of approximately 20 ng as input for the assay. For sequencing, prepared libraries were loaded according to the manufacturer’s instructions into Ion 540^TM^ Chips (Thermofisher Scientific, Waltham, MA, USA) and prepared using the Ion Chef^TM^ System (Thermofisher Scientific, Waltham, MA, USA). Sequencing was performed using the Ion S5^TM^ Sequencer (Thermofisher Scientific, Waltham, MA, USA). Data were mapped to the human genome assembly, embedded as the standard reference genome in the Ion Reported^TM^ Software version 5.18 (Thermofisher Scientific, Waltham, MA, USA). We used the Ion Reported^TM^ Software for initial automated analysis. Moreover, coverage analysis reported from the Ion Reported^TM^ Software, providing measurements of mapped reads, mean depth, uniformity, and alignment over a target region, was used as a quality assessment of the sequencing reactions. Indeed, formalin caused the deamination of cytosine, producing a base substitution of cytosine to thiamine or guanosine to adenosine (on the antisense strand), thereby generating irreversible false-positive sequencing artefactual variants that may compromise the interpretation of low-frequency variants. Files for analysis were provided by the Ion Reported^TM^ Software with the utilization of data interchange standard parameters defined via a JavaScript Object Notation file. Files were downloaded without any filter chain, providing all identified variants.

The analysis of obtained data predicted: DNA mutational analysis, for the identification of variants with coding effect and, also, of splicing region and untranslated region, UTR (region located or upstream or downstream in the coding sequence gene, that could change the protein synthesize); DNA copy number variation analysis, for the identification of genes amplification condition and RNA transcripts analysis for the identification of fusion genes.

Molecular data interpretation comprised three principal phases. During the first phase, all variants that were not qualitatively unsuitable were excluded. This filtering was performed using a coverage and Phred score, based on parameters suggested by Vestergaard L.K. et al. in their study [63]. Particularly, they had coverage > 100×, a Phred score > 200, and a *p*-value < 0.01. During the second phase, the filtering regarded the benign population polymorphisms. They were defined as mutations, commonly presented in the general population with a frequency greater than 1%. Indeed, they were generally associated with individual variability and were not usually associated with pathogenic activity. Screening of the benign population polymorphisms was performed by consulting the principal genome databases, such as the University of California, Santa Cruz (UCSC) Genome Browser, the Genome Aggregation Database Browser (GNOMAD), and the 1000 Genome/Ensembl Browser. Subsequently, a third phase was necessary to identify the pathogenic classes of the variants. In fact, using the American College of Medical Genetics and Genomics (ACMG) guidelines, several mutations were classified as pathogenic (pa), likely pathogenic (lp), VUS, likely benign, and benign. The subdivision was made by consulting several databases, such as VarSome [64], Franklin [65], CancerVar/ClinVar [66], and cBioPortal [67].

Finally, for the subsequent analyses, qualitatively suitable variants were kept, which were not known as population polymorphisms, regardless of the pathogenic classification. Notably, it was not possible to certainly distinguish the origin somatic and germinal of the variants because it was necessary to test in parallel the patients’ genomic DNA in healthy tissue or in peripheral blood.

### 4.4. Statistical Analysis

All analyses were performed within a Bayesian inferential framework using R (version 4.3.2) and Stan (version 2.32.2).

Descriptive statistics were computed for all variables analyzed: categorical variables were described using absolute and relative frequencies, whereas numerical features were reported as mean and standard deviation, or median and interquartile range.

We compared genetic mutations and patients’ characteristics in different groups:

- Metastatic patients at onset or patients who relapsed within 6 months after the end of adjuvant treatment (group M) versus relapsed after this time (group R).
- Old patients with more than the median age population (72 years old) (O) versus younger ones (Y).
- Endometrioid histology (E) versus non-endometrioid histology (N); this latter group included serous carcinoma, cell clear carcinoma, and undifferentiated carcinoma
- Overweight and obese patients (W) with a Body Mass Index (BMI) greater than 24.9 versus skinny ones (S).

To perform these comparisons, univariable Bayesian logistic regression models were fitted, and the results were expressed in terms of log-odds ratios.

Overall survival (OS) was analyzed using univariable and multivariable Bayesian Weibull regression models. For each candidate covariate, a separate model was fitted to quantify its association with survival time, and the distribution of regression coefficients (β) was reported.

The time-to-event variable was defined as the interval between the date of diagnosis of metastatic disease or relapse and death or censoring. Due to sample size limitations, the multivariable model only included a limited set of covariates, selected based on the results obtained from univariable models.

Posterior distributions were obtained using four independent Markov Chain Monte Carlo (MCMC) chains with 3000 iterations each (1000 warm-up), with convergence verified through visual inspection of trace plots and the Gelman–Rubin statistic. Posterior estimates were reported as posterior means or medians, and 90% credible intervals (CI). Also, each association was reported with the probability of direction (PD) and the Region of Practical Equivalence (ROPE).

## 5. Conclusions

Our exploratory analysis, despite the inherent limitations of its small sample size and retrospective design, delineated the mutational landscape of a high-risk EC population and assessed the association between selected genomic alterations and specific clinical and tumor characteristics. Studies of this nature may support clinicians in prioritizing targeted molecular testing—particularly in settings where comprehensive next-generation sequencing panels are not feasible—and in refining therapeutic decision-making. Although artificial intelligence-based approaches may in the future facilitate the identification of molecular patterns from radiological imaging, the integration of clinical characteristics with established knowledge of tumor molecular profiles remains fundamental for the appropriate selection and interpretation of molecular analyses and for the translation of genomic data into diagnostic and therapeutic strategies. Within this framework, in our exploratory work, we observed that *KRAS* and *CTNNB1* mutations were associated with differential survival outcomes, underscoring their potential biological and clinical relevance in EC. The small sample size, retrospective nature of our trial, and heterogeneous composition and treatment history of our cohort limit definitive conclusions and offer only preliminary findings. Further investigations through translational research are warranted. This includes functional studies to elucidate the role of *KRAS*- and *CTNNB1*-driven pathways in disease progression and treatment response, as well as larger, prospective, biomarker-driven clinical trials evaluating molecularly guided and combination therapeutic approaches.

Given the complexity and dynamic nature of the EC molecular landscape, continued efforts to integrate existing evidence with emerging data are required to clarify interactions among co-occurring molecular alterations and to define their clinical utility, ultimately improving personalized patient management.

## Figures and Tables

**Table 1:** Patient characteristics (*N* = 29) °.

Characteristic	Value
Age at diagnosis, years, median (range)	70 (50–86)
Age at relapse or metastatic disease, years, median (range)	72 (50–87)
Body Mass Index (BMI), kg/m^2^ *, median (range)	27.6 (18.7–37.1)
Surgery performed	24 (82.8%)
Disease status	
- Metastatic at diagnosis or relapse ≤ 6 months (M)	14 (48.3%)
- Relapse > 6 months from diagnosis (R)	15 (51.7%)
Age groups	
- Older than median age (72 years) (O)	15 (51.7%)
- Younger than median age (Y)	14 (48.3%)
Histology	
- Endometrioid (E)	17 (58.6%)
- Non-endometrioid (N)	12 (41.4%)
BMI categories *	
- Overweight and obese (W)	14 (56.0%)
- Normal weight or underweight (S)	11 (44.0%)
Alive/death	6/23

° Characteristics refer to the 29 patients with available mutational DNA analysis. * BMI data available for 25 patients.

**Table 2:** Selected gene mutations in the molecular landscape of advanced EC.

Gene	Key Findings	Clinical-Biological Significance
*ARID1A* [42]	Mutations predominantly occur in endometrioid histology	Loss of protein expression is associated with poor prognosis and genomic instability in several cancers; evidence in EC remains conflicting
*CCND1* [43]	Over-expression in the early-stage endometrioid EC; reduced expression in high-grade subtypes	Plays a key role in EC progression and recurrence
*CTNNB1* [44]	Mutations are particularly frequent in the NSMP molecular subgroup	Associated with recurrence in early-stage endometrioid EC, especially within NSMP
*FBXW7* [45]	Mutation status correlates with tumor grade and EC histotype	Linked to poor prognosis, potentially through increased treatment resistance
*FGFR2* [46]	More frequent in patients diagnosed with stage III/IV EC compared with stage I/II	Associated with shorter progression-free and EC-specific survival
*KRAS* [40]	Relatively frequent alterations in EC (16% of cases)	Overlaps with genomic predictors of response to immunotherapy
*PIK3CA* [28]	Frequent alterations affecting the PI3K signaling pathway in EC	Lead to pathway activation and represent a potential target for therapy
*PPP2R1A* [47]	Mutations associated with non-localized disease and non-endometrioid histology	Predict high-risk features, but they are not independently prognostic in EC
*PTEN* [48,49]	Frequently co-occurs with PIK3CA mutations in EC	May increase tumor sensitivity to PI3K/AKT/mTOR inhibitors
*TP53* [50]	Associated with advanced stage, non-endometrioid histology, and high-grade EC	Negative prognostic factor

**Table 3:** Distribution of selected filtered DNA mutations in the overall cohort, group M and group R.

Gene	Mutation Status	All (*N* = 29)	Group M (*N* = 14)	Group R (*N* = 15)
*ARID1A*	No mutation/VUS	25 (86.2%)	12 (85.7%)	13 (86.7%)
	lp/pa	4 (13.8%)	2 (14.3%)	2 (13.3%)
*CCND1*	No mutation/VUS	26 (89.7%)	12 (85.7%)	14 (93.3%)
	lp/pa	3 (10.3%)	2 (14.3%)	1 (6.7%)
*CTNNB1*	No mutation/VUS	24 (82.8%)	13 (92.9%)	11 (73.3%)
	lp/pa	5 (17.2%)	1 (7.1%)	4 (26.7%)
*FBXW7*	No mutation/VUS	27 (93.1%)	12 (85.7%)	15 (100.0%)
	lp/pa	2 (6.9%)	2 (14.3%)	0 (0.0%)
*FGFR2*	No mutation/VUS	24 (82.8%)	11 (78.6%)	13 (86.7%)
	lp/pa	5 (17.2%)	3 (21.4%)	2 (13.3%)
*KRAS*	No mutation/VUS	23 (79.3%)	11 (78.6%)	12 (80.0%)
	lp/pa	6 (20.7%)	3 (21.4%)	3 (20.0%)
*PIK3CA*	No mutation/VUS	18 (62.1%)	7 (50.0%)	11 (73.3%)
	lp/pa	11 (37.9%)	7 (50.0%)	4 (26.7%)
*PPP2R1A*	No mutation/VUS	25 (86.2%)	12 (85.7%)	13 (86.7%)
	lp/pa	4 (13.8%)	2 (14.3%)	2 (13.3%)
*PTEN*	No mutation/VUS	14 (48.3%)	8 (57.1%)	6 (40.0%)
	lp/pa	15 (51.7%)	6 (42.9%)	9 (60.0%)
*TP53*	No mutation/VUS	23 (79.3%)	12 (85.7%)	11 (73.3%)
	lp/pa	6 (20.7%)	2 (14.3%)	4 (26.7%)

Legend: No mutation/VUS: patients with no detected alterations or with benign, likely benign, or variants of uncertain significance (VUS) alterations; lp/pa: patients harboring likely pathogenic or pathogenic mutations.

## Data Availability

The raw data supporting the conclusions of this article will be made available by the authors on request.

## Supplementary Materials

The following supporting information can be downloaded at: https://www.mdpi.com/article/10.3390/ijms27021096/s1.

## Abbreviations

The following abbreviations are used in this manuscript:

EC	Endometrial Cancer
HNPCC	Non-polyposis colorectal cancer syndrome
WHO	World Health Organization
TCGA	The Cancer Genome Atlas
ProMisE	Proactive Molecular Risk Classifier for Endometrial Cancer
dMMR	Mismatch repair-deficient
MSI-H	Microsatellite instability-high
NSMP	No specific molecular profile
ER	Estrogen receptor
IHC	Immunohistochemical
L1CAM	L1 cell adhesion molecule
CTNNB1	β-catenin 1
KRAS	Kirsten Rat Sarcoma virus
PI3K	Phosphatidylinositol 3-Kinases
PIK3CA	Phosphatidylinositol 3-kinase catalytic subunit alpha
ARID1A	AT-Rich Interactive Domain-containing protein 1A
PFS	Progression-free survival
OS	Overall survival
ORR	Objective Response Rate
FDA	Food and Drug Administration
EMA	European Medicines Agency
pa	Pathogenic
lp	Likely pathogenic
VUS	Variant of Uncertain Significance
mut	Mutations
CNV	Copy Number Variation
MAPD	Median Absolute Pairwise Difference
PD	Probability of direction
ROPE	Region of Practical Equivalence
OR	Odds ratio
BMI	Body Mass Index
mAF	median allele frequency
ACMG	American College of Medical Genetics and Genomics
vs	versus
N	Non-endometrioid Histology
E	Endometrioid histology
M	Metastatic at diagnosis or relapse within 6 months
R	Relapse after 6 months from diagnosis
W	Overweight and obese
S	Normal weight or underweight
O	Older than the median age
Y	Younger than the median age
