# Supplementary material for: Molecular Landscape of Advanced Endometrial Cancer: Exploratory Analyses at Modena Cancer Center (MEMO)
Source: Int J Mol Sci. 2026 Jan 22;27(2):1096. doi: 10.3390/ijms27021096 (PMC12842487; doi:10.3390/ijms27021096)
Supplement: Supplementary file 1 [file ijms-27-01096-s001.zip › ijms-4009611-supplementary.pdf]

Supplementary Material

Article

Molecular Landscape of Advanced Endometrial Cancer: Exploratory Analyses at Modena Cancer Center (MEMO)

Marta Pirola [1,†], Eleonora Molinaro [2,†], Samantha Manfredini [3], Riccardo Cuoghi Costantini [4], Chiara Carlucci [1], Claudia Piombino [5], Stefania Pipitone [5], Maria Giuseppa Vitale [5], Roberto Sabbatini [5], Francesca Bacchelli [6], Laura Botticelli [7], Albino Eccher [7], Roberto D'Amico [4], Lucia Longo [2], Stefania Bettelli [3], Cinzia Baldessari [5,*,‡] and Massimo Dominici [1,5,‡]

[1] Division of Oncology, University of Modena and Reggio Emilia, 41124 Modena, Italy; marta.pirola04@gmail.com (M.P.); 325498@studenti.unimore.it (C.C.); mdominici@unimore.it (M.D.)
[2] Oncology Area Sud AUSL Modena, Ospedale of Sassuolo, 41049 Sassuolo, Italy; e.molinaro@ausl.mo.it (E.M.); l.longo@ausl.mo.it (L.L.)
[3] Division of Molecular Pathology and Predictive Medicine, Azienda Ospedaliero-Universitaria of Modena, 41124 Modena, Italy; manfredini.samantha@aou.mo.it (S.M.); bettelli.stefania@aou.mo.it (S.B.)
[4] Statistics Unit, Department of Diagnostic and Clinical Medicine and Public Health, University of Modena and Reggio Emilia, 41124 Modena, Italy; 337988@studenti.unimore.it (R.C.C.); roberto.damico@unimore.it (R.D.)
[5] Division of Oncology, Department of Oncology & Hematology, Azienda Ospedaliero-Universitaria of Modena, 41124 Modena, Italy; claudia.piombino@outlook.com (C.P.); pipitone.stefania@aou.mo.it (S.P.); vitale.mariagiuseppa@aou.mo.it (M.G.V.); sabbrob@unimore.it (R.S.)
[6] Clinical Trials Office, Division of Oncology, University of Modena and Reggio Emilia, 41124 Modena, Italy; francesca.bacchelli@unimore.it
[7] Department of Pathology, Azienda Ospedaliero-Universitaria of Modena, 41124 Modena, Italy; botticelli.laura@aou.mo.it (L.B.); eccher.albino@aou.mo.it (A.E.)
* Correspondence: baldessari.cinzia@aou.mo.it
† These authors contributed equally to this study.
‡ These authors also contributed equally to this study.

Supplemental Table S1. Association between patient groups and clinical and genetic variables.

			Distribution of log (OR)						
**Group**	**Variable**	**Comparison**	**Mean**	**SD**	**5%**	**50%**	**95%**	**ROPE**	**PD**
W vs S	*ARID1A*	lp or pa vs others	-0,297	1,169	-2,26	-0,27	1,65	0,130	0,589
W vs S	*CTNNB1*	lp or pa vs others	-0,875	1,116	-2,73	-0,82	0,90	0,098	0,800
W vs S	*FGFR2*	lp or pa vs others	-1,318	1,543	-3,91	-1,19	1,10	0,070	0,803
W vs S	*KRAS*	lp or pa vs others	0,288	1,074	-1,49	0,19	2,08	0,148	0,578
W vs S	*PIK3CA*	lp or pa vs others	-1,161	0,848	-2,64	-1,13	0,26	0,078	0,906
W vs S	*PPP2R1A*	lp or pa vs others	1,312	1,422	-0,73	1,27	3,84	0,072	0,841
W vs S	*PTEN*	lp or pa vs others	-0,109	0,834	-1,49	-0,08	1,30	0,183	0,540
W vs S	*TP53*	lp or pa vs others	0,676	1,071	-0,88	0,69	2,52	0,123	0,753
W vs S	N vs E	N vs E	1,360	0,904	-0,04	1,36	2,87	0,047	0,947
W vs S	O vs Y	O vs Y	-0,539	0,852	-1,86	-0,49	0,82	0,163	0,722
W vs S	age at rel/met	1-unit increase	0,035	0,047	-0,04	0,03	0,12	1,000	0,759
O vs Y	*ARID1A*	lp or pa vs others	-1,629	1,405	-4,28	-1,50	0,45	0,062	0,894
O vs Y	*CTNNB1*	lp or pa vs others	0,484	1,086	-1,23	0,44	2,26	0,140	0,668
O vs Y	*FGFR2*	lp or pa vs others	1,917	1,375	-0,11	1,80	4,44	0,046	0,937
O vs Y	*KRAS*	lp or pa vs others	-2,444	1,361	-4,57	-2,18	-0,38	0,008	0,982
O vs Y	*PIK3CA*	lp or pa vs others	-1,758	0,893	-3,31	-1,73	-0,40	0,000	0,987
O vs Y	*PPP2R1A*	lp or pa vs others	-1,697	1,427	-4,06	-1,52	0,37	0,061	0,902
O vs Y	*PTEN*	lp or pa vs others	1,322	0,829	0,07	1,33	2,68	0,044	0,963
O vs Y	*TP53*	lp or pa vs others	-1,090	1,026	-2,77	-1,01	0,55	0,090	0,849
O vs Y	W vs S	W vs S	-0,500	0,831	-1,85	-0,46	0,87	0,159	0,714
O vs Y	N vs E	N vs E	-0,140	0,765	-1,40	-0,12	1,13	0,197	0,560
O vs Y	BMI	1-unit increase	0,062	0,074	-0,06	0,06	0,19	0,967	0,801
R vs M	*ARID1A*	lp or pa vs others	-0,057	1,201	-2,02	-0,06	1,90	0,126	0,520
R vs M	*CTNNB1*	lp or pa vs others	1,931	1,342	-0,18	1,75	4,43	0,050	0,930
R vs M	*FGFR2*	lp or pa vs others	-0,678	1,087	-2,49	-0,66	1,02	0,119	0,740
R vs M	*KRAS*	lp or pa vs others	-0,089	0,950	-1,62	-0,09	1,52	0,161	0,538
R vs M	*PIK3CA*	lp or pa vs others	-1,062	0,818	-2,46	-1,06	0,21	0,075	0,918
R vs M	*PPP2R1A*	lp or pa vs others	-0,044	1,192	-2,08	-0,13	1,75	0,138	0,548
R vs M	*PTEN*	lp or pa vs others	0,747	0,765	-0,55	0,72	2,00	0,122	0,826
R vs M	*TP53*	lp or pa vs others	0,934	1,054	-0,78	0,85	2,65	0,110	0,795
R vs M	W vs S	W vs S	1,530	0,890	0,15	1,54	3,06	0,029	0,963
R vs M	N vs E	N vs E	-0,735	0,759	-1,99	-0,70	0,54	0,124	0,829
R vs M	O vs Y	O vs Y	0,146	0,759	-1,14	0,10	1,38	0,191	0,554
R vs M	age at rel/met	1-unit increase	0,043	0,042	-0,02	0,04	0,12	1,000	0,857
R vs M	BMI	1-unit increase	0,212	0,091	0,07	0,21	0,37	0,386	0,996
N vs E	*ARID1A*	lp or pa vs others	-1,202	1,433	-3,80	-1,10	0,94	0,091	0,792
N vs E	*CTNNB1*	lp or pa vs others	-1,510	1,333	-3,87	-1,41	0,42	0,063	0,896
N vs E	*FGFR2*	lp or pa vs others	-1,571	1,364	-3,87	-1,38	0,47	0,068	0,890
N vs E	*KRAS*	lp or pa vs others	-1,835	1,307	-4,20	-1,68	0,06	0,052	0,942
N vs E	*PIK3CA*	lp or pa vs others	0,275	0,788	-1,03	0,29	1,63	0,175	0,635
N vs E	*PPP2R1A*	lp or pa vs others	1,983	1,412	-0,09	1,88	4,57	0,045	0,939
N vs E	*PTEN*	lp or pa vs others	-1,359	0,845	-2,78	-1,38	-0,12	0,038	0,962

N vs E	*TP53*	lp or pa vs others	1,452	1,037	-0,18	1,39	3,14	0,058	0,927
N vs E	W vs S	W vs S	1,339	0,895	-0,03	1,33	2,76	0,056	0,945
N vs E	O vs Y	O vs Y	-0,098	0,759	-1,39	-0,12	1,15	0,194	0,565
N vs E	age at rel/met	1-unit increase	0,011	0,042	-0,06	0,01	0,08	1,000	0,591
N vs E	BMI	1-unit increase	0,041	0,075	-0,08	0,04	0,17	0,993	0,704

Abbreviations: OR: odds ratio; SD: Standard deviation; ROPE: region of practical equivalence; PD: probability of direction; BMI: body mass index; N: Non-endometrioid Histology; E: endometrioid histology; M: Metastatic at onset or relapsed within 6 months; R: Relapse after 6 months; W: Overweight and obese patients; S: Normal weight or underweight; O: Older than median age; Y: Younger than median age; lp: likely pathogenic mutations; pa: pathogenic mutations; age at rel/met: age at relapsed or diagnosis of metastatic disease.

Supplemental Table S2. Association between patient groups and genetic mutations with respect to survival probability.

Distribution of Weibull models coefficients									
**Variabile**	**Comparison**	**Coef.**	**mean**	**SD**	**5%**	**50%**	**95%**	**ROPE**	**PD**
ARID1A	lp or pa vs others	beta	0,047	0,792	-1,16	-0,01	1,47	0,115	0,505
ARID1A		alpha	0,752	0,123	0,56	0,75	0,96	0,000	1,000
CTNNB1	lp or pa vs others	beta	1,485	0,866	0,17	1,42	3,01	0,015	0,972
CTNNB1		alpha	0,775	0,124	0,58	0,77	0,98	0,000	1,000
FGFR2	lp or pa vs others	beta	0,384	0,694	-0,69	0,36	1,58	0,112	0,708
FGFR2		alpha	0,755	0,124	0,56	0,75	0,97	0,000	1,000
KRAS	lp or pa vs others	beta	-1,669	0,596	-2,54	-1,72	-0,62	0,000	0,993
KRAS		alpha	0,913	0,157	0,67	0,91	1,18	0,000	1,000
PIK3CA	lp or pa vs others	beta	0,118	0,547	-0,76	0,12	1,02	0,148	0,584
PIK3CA		alpha	0,757	0,126	0,56	0,75	0,97	0,000	1,000
PPP2R1A	lp or pa vs others	beta	-0,896	0,715	-1,99	-0,95	0,34	0,050	0,892
PPP2R1A		alpha	0,787	0,130	0,58	0,78	1,01	0,000	1,000
PTEN	lp or pa vs others	beta	0,586	0,564	-0,31	0,57	1,52	0,089	0,859
PTEN		alpha	0,756	0,123	0,56	0,75	0,96	0,000	1,000
TP53	lp or pa vs others	beta	0,275	0,663	-0,76	0,26	1,40	0,126	0,656
TP53		alpha	0,754	0,125	0,56	0,75	0,97	0,000	1,000
W vs S	W vs S	beta	0,748	0,602	-0,21	0,74	1,75	0,061	0,901
W vs S		alpha	0,757	0,138	0,54	0,75	0,99	0,000	1,000
N vs E	N vs E	beta	0,558	0,563	-0,33	0,54	1,50	0,089	0,853
N vs E		alpha	0,758	0,126	0,56	0,75	0,97	0,000	1,000
O vs Y	O vs Y	beta	-0,108	0,544	-0,97	-0,12	0,79	0,150	0,587
O vs Y		alpha	0,755	0,125	0,56	0,75	0,97	0,000	1,000
R vs M	R vs M	beta	0,854	0,540	-0,01	0,84	1,79	0,042	0,948
R vs M		alpha	0,764	0,127	0,57	0,76	0,98	0,000	1,000

Abbreviations: OR: odds ratio; SD: Standard deviation; ROPE: region of practical equivalence; PD: probability of direction; BMI: body mass index; N: Non-endometrioid Histology; E: endometrioid histology; M: Metastatic at onset or relapsed within 6 months; R: Relapse after 6 months; W: Overweight and obese patients; S: Normal weight or underweight; O: Older than median age; Y: Younger than median age; lp: likely pathogenic mutations; pa: pathogenic mutations; age at rel/met: age at relapsed or diagnosis of metastatic disease.
